# Reduced Shear Stress and Longer Blood Flow Time Occur in Both Severe Focal and Mild Diffuse LAD Lesions: Angiograms Alone Don’t Always Reveal Their True Impact on Blood Flow

**DOI:** 10.3390/pathophysiology32020028

**Published:** 2025-06-19

**Authors:** Gianluca Rigatelli, Marco Zuin, Niva Mileva, Dobrin Vassilev, Giuseppe Marchese, Ervis Hiso, Andrea Bertolini, Claudio Bilato

**Affiliations:** 1 Interventional Cardiology Unit, Madre Teresa di Calcutta Hospitals, Padova South Hospitals, 35043 Monselice, Italy; marco.zuin@aulss6.veneto.it (M.Z.); giuseppe.marchese@aulss6.veneto.it (G.M.); ervis.hiso@aulss6.veneto.it (E.H.); andrea.bertolini@aulss6.veneto.it (A.B.); 2 Department of Translational Medicine, University of Ferrara, 44124 Ferrara, Italy; 3 Department of Cardio-Thoraco-Vascular Sciences and Public Health, University of Padova, 35100 Padua, Italy; 4 Medica Cor Hospital, 1713 Ruse, Bulgaria; nmileva91@gmail.com (N.M.); dobrinv@gmail.com (D.V.); 5 Department of Cardiology, West Vicenza Hospitals, 36071 Arzignano, Italy; claudio.bilato@aulss6.veneto.it

**Keywords:** coronary artery disease, fluid dynamic, rheology, computation fluid dynamic

## Abstract

**Background/Objectives:** The similarities and differences from a rheological perspective between significant short focal and mild long coronary lesions warrant investigation to elucidate wall shear stress (WSS) angiographic discrepancies. **Methods**: Patients who underwent coronary computed tomography angiography (CCTA) between 1 January 2023 and 1 September 2024 were selected for computational fluid dynamics (CFD) analysis. The selection criteria included either a focal (≤20 mm) hemodynamically significant stenosis, defined as ≥75% lumen narrowing, or a long (30–40 mm) non-hemodynamically significant lesion showing ≤50% stenosis of the left anterior descending (LAD) artery. Patient-specific models were reconstructed from ECG-gated CCTA images. Wall shear stress (WSS, measured in Pascals) and residence time (RT) were evaluated for each patient. **Results**: The LAD arteries of 30 patients (mean age 54 years, 63.3% men) were evaluated: 16 with focal, hemodynamically significant coronary stenosis, while 14 with diffuse, long, non-hemodynamically significant coronary lesions. Both groups exhibited a lower mean WSS compared to the non-stenosed segment, with no significant difference in mean WSS between the two groups (*p* = 0.84). Conversely, both groups demonstrated a higher mean residence time (RT) compared to the non-stenosed segments (0.2 ± 0.06 vs. 0.60 ± 0.03, *p* < 0.001 and 0.2 ± 0.006 vs. 0.59 ± 0.02, *p* < 0.001, respectively), and no significant difference in mean RT (*p* = 0.82). **Conclusions**: Long, angiographically mild coronary stenoses show similar WSS and RT characteristics compared to short hemodynamically significant coronary stenosis.

## 1. Introduction

In clinical practice, coronary lesion with at least 70% diameter stenosis on coronary angiography was considered significant [1]. However, over the last decade, the advent of invasive functional assessment using fractional flow reserve (FFR) has demonstrated that long coronary stenoses can yield positive FFR results despite appearing angiographically insignificant [2,3]. Intravascular ultrasound imaging studies have identified a significant correlation between minimal lumen area and FFR [4], while other imaging techniques have found local geometry, including lesion length, to be a predictor of low FFR values [5], suggesting a role of Poiseuille’s law in limiting coronary flow [6]. The FAME trials highlighted the discrepancies between angiography and FFR [5]. Angiography provides a two-dimensional evaluation of coronary lesions, while FFR measures lesion-induced ischemia, which depends not only on the percent reduction in lumen area but also on parameters like lesion length, longitudinal eccentricity, cross-sectional eccentricity, surface roughness, and proximal location [5].

Specifically, Poiseuille’s law, which describes the relationship between flow, vessel radius, and length, indicates that even small reductions in lumen diameter or increases in lesion length can markedly impair flow. The rheological similarities and differences between significant short focal and mild long coronary lesions have not been investigated. In this context, this study aimed to compare local wall shear stress (WSS) and residence time (RT) between patients with focal, hemodynamically significant coronary stenosis and those with long, non-hemodynamically significant coronary lesions in a prognostic vessel such as the left anterior descending (LAD) artery.

## 2. Materials and Methods

### 2.1. Study Population

Between 1 January 2023 and 1 September 2024, consecutive patients who underwent CCTA for any reason were included if they have (i) a focal (length ≤ 20 mm) hemodynamically significant stenosis, defined as a narrowing of the lumen ≥ 75% or a (ii) long (length ≥ 30 mm ≤ 40 mm) non-hemodynamically significant lesions showing a stenosis ≤ 50% of the LAD. On the other hand, the exclusion criteria included (i) lesions affecting the left anterior descending (LAD) artery ostium, (ii) stenoses in other major coronary vessels, (iii) history of coronary stenting or bypass surgery, (iv) significant motion or blurring artifacts in CCTA images, (v) incomplete clinical or imaging data, (vi) non-diagnostic image quality, and (vii) advanced chronic kidney disease. Thus, the final study population comprised 30 patients, of which 16 had a focal hemodynamically significant lesion and 14 had a diffuse–long non-hemodynamically significant stenosis of the LAD. Demographic information, including patient age, sex, body mass index (BMI), smoking status, and history of arterial hypertension and diabetes mellitus, was systematically collected for all participants. Calculated low-density lipoprotein cholesterol (C-LDL) was derived using the Friedwald formula. The study was conducted in accordance with the Declaration of Helsinki and approved by the local Ethics Committees. Due to the retrospective nature of this analysis, the requirement for written informed consent was waived.

### 2.2. CCTA Protocol

Cardiac CT angiography (CCTA) images were obtained using an ECG-gated 128-slice CT scanner (Somatom Definition Flash, Siemens, Germany), following the guidelines established by the Society of Cardiovascular Computed Tomography [7]. The standard CCTA protocol included an initial calcium scoring scan to define the scanning range, extending from the tracheal carina to the diaphragm. Based on the patient’s body weight, 60–80 mL of iodinated contrast agent (Iomeron 350 mg/mL, Bracco) was administered via a cubital vein using a dual-head injector at a flow rate of 5.0 mL/s, followed by a 30 mL saline flush. A bolus tracking method was employed at the level of the aortic root to determine the appropriate timing for image acquisition. Once the contrast density reached 90 Hounsfield units (HU), the scan commenced automatically after a 6 s delay, during a single breath-hold and concurrent ECG recording. The image parameters were slice collimation of 32 × 0.6 mm and slice acquisition of 128 × 0.6 mm by means of a z-axis flying focal spot, 0.33 s rotation time, with an 83 ms temporal resolution. Images were reconstructed from the raw data at 5% intervals throughout the entire RR interval (ranging from 5% to 100%), using a slice thickness of 0.75 mm, a reconstruction increment of 0.5 mm, and a B26f reconstruction kernel.

### 2.3. Computational Fluid Dynamics

Patient-specific anatomical models were generated from ECG-gated CCTA images using crOsiriX software (OsiriX Foundation, Geneva, Switzerland) (https://www.osirix-viewer.com). These models were then refined using Rhinoceros version 4.0 (McNeel & Associates, Indianapolis, IN, USA), following established protocols [8]. The geometries were discretized in Ansys ICEM CFD (ANSYS Inc., Canonsburg, PA, USA) into linear tetrahedral elements, with a maximum surface element size of 0.2 mm and a global maximal wall-face edge size of 0.15 mm. Prior to CFD simulation, a mesh independence analysis was conducted using the region adaptation tool in FLUENT to ensure further mesh refinement did not significantly affect the results. A four-layer prism mesh with a height ratio of 1.2 was applied to accurately resolve near-wall flow dynamics [9]. The final mesh consisted of 300,152 elements and 52,962 nodes. Blood was modeled as a non-Newtonian, viscous, incompressible fluid with a density of 1060 kg/m^3^, in line with standard references [10]. Flow dynamics were governed by the Navier–Stokes and continuity equations [11], with viscosity behavior described using the Carreau model [12]. Given that coronary perfusion predominantly occurs during diastole, a steady-state flow was assumed at the coronary ostia inlet [13]. Patient-specific stroke volume was calculated as the difference between end-diastolic (EDV) and end-systolic volume (ESV) and combined with heart rate (HR) to derive the mean flow rate. The corresponding inlet velocity profile was applied at the left main coronary ostium, while flow distribution at the outlets (LAD and LCX) was defined based on Murray’s law, which assumes flow distribution proportional to the cube of the branch diameters [14]. Coronary stenosis severity (DS%) and minimal lumen area were quantified using the reconstructed coronary geometry. Stenosis severity was calculated as 100% minus the ratio of the minimal lumen area to the reference area, expressed as a percentage [15]. Wall shear stress (WSS), expressed in Pascals (Pa), was defined as the tangential force exerted by blood flow on the vascular endothelium [16]. Residence time (RT), a hemodynamic parameter indicative of regions at risk for restenosis due to low WSS and flow stagnation, was assessed as described by Hashemi et al. [17] using velocity vectors. To evaluate WSS and RT, for each lesion, we defined a segment that included the stenotic region as well as the adjacent proximal and distal vessel segments. Within these regions, we computed time-averaged WSS and residence time values across the entire segment rather than isolating only the point of maximal stenosis. This approach allowed us to characterize the overall hemodynamic profile of the lesion and its surrounding environment. The segmental averages were then used for statistical comparisons between groups. All simulations were conducted using ANSYS FLUENT 14.0 (ANSYS Inc.).

### 2.4. Statistical Analysis

Continuous variables following a normal distribution are reported as mean ± standard deviation (SD), whereas those with a non-normal distribution are expressed as median with interquartile range (IQR). Categorical variables are presented as percentages. The chi-squared test was employed to compare categorical data, while continuous variables were analyzed using either Student’s *t*-test or the Mann–Whitney U test, depending on data distribution. A *p*-value less than 0.05 was considered statistically significant. All statistical analyses were conducted using R software (R Foundation for Statistical Computing, Vienna, Austria).

## 3. Results

### 3.1. General Population

The left anterior descending (LAD) arteries of 30 patients (mean age 54 years, 63.3% men) were evaluated. Sixteen patients presented with focal, hemodynamically significant coronary stenosis, while fourteen exhibited diffuse, long, non-hemodynamically significant coronary lesions affecting the LAD. The baseline demographic and clinical characteristics of the study population are provided in Table 1.

### 3.2. WSS

**Figure 1:**
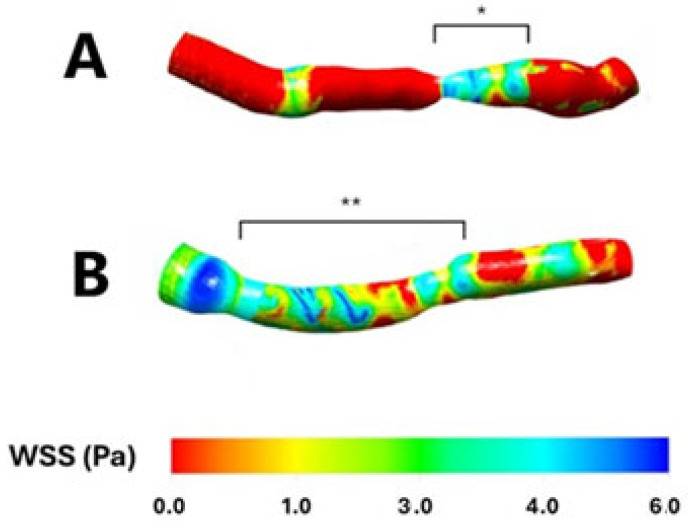
Wall shear stress in two sample patients having a focal, hemodynamically significant left anterior descending stenosis (Panel (**A**); *) and a diffuse, long, non-hemodynamically significant left anterior descending stenosis (Panel (**B**); **), respectively. WSS: Wall shear stress. Pa: Pascal.

**Figure 2:**
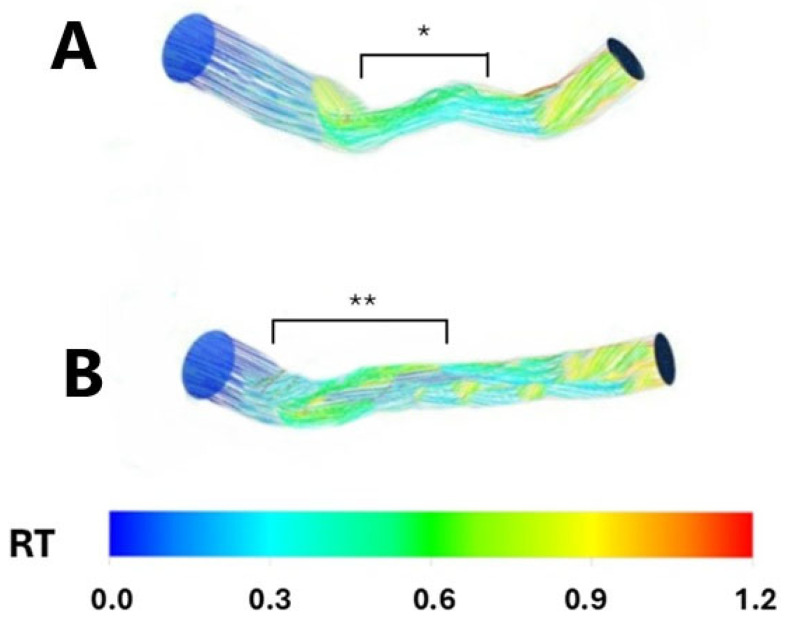
Resident time in two sample patients having a focal, hemodynamically significant left anterior descending stenosis (Panel (**A**); *) and a diffuse, long, non-hemodynamically significant left anterior descending stenosis (Panel (**B**); **), respectively. RT: Resident time.

Both patients with focal, hemodynamically significant LAD stenosis and those with diffuse, long, non-hemodynamically significant LAD lesions exhibited significantly lower mean wall shear stress (WSS) compared to non-stenosed segments (3.62 ± 0.4 vs. 1.8 ± 0.2, *p* < 0.001, and 3.6 ± 0.3 vs. 1.9 ± 0.3, *p* < 0.001, respectively). However, no significant difference in mean WSS was observed when comparing the focal, hemodynamically significant stenosed segment with the diffuse, long, non-hemodynamically significant stenosed segments (*p* = 0.84), corresponding to areas of blood flow recirculation (Figure 1 and Figure 2).

### 3.3. RT

**Figure 3:**
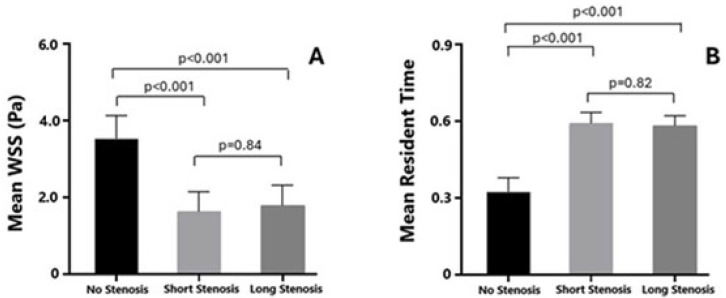
Comparison of mean wall shear stress (Panel (**A**)) and resident time (Panel (**B**)) between left anterior descending segments having a focal, hemodynamically significant, diffuse–long, non-hemodynamically significant stenosis and healthy left anterior descending segment.

Conversely, patients with both focal; hemodynamically significant; and diffuse, long, non-hemodynamically significant LAD stenosis demonstrated a higher mean residence time (RT) compared to the non-stenosed segments (0.2 ± 0.06 vs. 0.60 ± 0.03, *p* < 0.001 and 0.2 ± 0.006 vs. 0.59 ± 0.02, *p* < 0.001, respectively). Similarly, no significant difference in mean RT was observed when comparing the focal, hemodynamically significant stenosed segments with the diffuse, long, non-hemodynamically significant stenosed segments (*p* = 0.82), indicating a prolonged period of coronary blood flow stagnation (Figure 2 and Figure 3).

## 4. Discussion

Our study demonstrated that long, non-hemodynamically significant LAD segments and focal, hemodynamically significant LAD segments share similar rheological characteristics in terms of wall shear stress (WSS) and residence time (RT). Specifically, both groups exhibited significantly lower mean WSS compared to normal LAD segments, reflecting areas of pronounced blood recirculation. Additionally, the similarly elevated RT observed in both lesion types highlights a comparable prothrombotic rheological environment, regardless of the degree of LAD stenosis. The observed difference seems to be less influenced by plaque features on coronary imaging, such as a plaque burden greater than 70%, a maximal lipid core burden index exceeding 315 in a 4 mm segment as measured by near-infrared spectroscopy, and the presence of thin-cap fibroatheroma, as shown in the PREVENT trial. In fact, the concept of a vulnerable plaque is distinct from that of an ischemia-causing lesion.

Therefore, the mechanisms explaining this discrepancy should be explored at a rheological level rather than at imaging or functional levels. CFD derived from patient-specific CCTA scans offers a unique tool to provide rheological information and compare significant short focal lesions versus mild, non-significant, long lesions [18]. The observation that stenosed arteries exhibited lower average WSS may seem counterintuitive, given that WSS typically peaks at the narrowest part of a lesion. However, our methodology considered the average hemodynamic values over the entire lesion segment, including proximal and distal areas, where flow separation and recirculation can result in significantly reduced WSS. These low-shear zones are thought to contribute more critically to atherogenic processes and plaque vulnerability than the localized high-shear stress at the stenosis throat. By analyzing segmental averages, our findings reflect the broader hemodynamic milieu rather than localized extremes, offering insights into how diffuse flow disturbances may influence vascular risk.

Our investigation revealed that short, focal, angiographically highly significant coronary stenoses share a similar rheological profile to long, angiographically mild coronary stenoses, not only in terms of WSS but also in terms of RT. This might explain why, from a functional point of view, the two subsets of lesions often share similar functional parameters [19]. Diffuse coronary artery disease lesions are well known to be longer and typically exhibit a greater degree of stenosis compared to focal lesions [20]. Furthermore, this difference in stenosis severity is a significant factor influencing physiological parameters such as FFR, which tends to be lower in diffuse lesions due to their more extensive involvement [21]. However, recent studies analyzing plaque phenotypes and coronary hemodynamics provide a more nuanced understanding. Using pressure pullback gradient to define focal versus diffuse disease, it was found that focal lesions, despite sometimes having less stenosis, are associated with a higher plaque burden, predominantly lipid-rich plaques, and a higher prevalence of thin-cap fibroatheroma, which are features linked to plaque vulnerability [21]. Conversely, diffuse lesions tend to have more calcified plaques and a more stable atherosclerotic phenotype [21]. This suggests that differences in lesion classification (focal vs. diffuse) reflect distinct pathophysiological processes beyond just stenosis severity. The focal lesions’ localized pressure gradients produce higher mechanical stress and flow disturbances, promoting plaque instability independent of stenosis severity. We observed no significant difference between the two focal/diffuse groups despite large differences in stenosis levels. Stenosis severity alone does not capture the complexity of lesion behavior and risk [22]. Therefore, while stenosis level is an important factor, the lesion being focal or diffuse encompasses additional biological and hemodynamic features that contribute to differences in clinical and physiological findings.

Our findings offer important clinical insights that challenge the traditional steno-sis-centric paradigm. The observation that both long, non-hemodynamically significant and short, hemodynamically significant LAD lesions exhibit similar adverse rheological profiles characterized by WSS and prolonged RT suggests that clinicians should broaden their interpretative focus beyond the degree of luminal narrowing. Additionally, present results emphasize the value of integrating lesion length and morphological context into the assessment rather than relying solely on stenosis severity or functional indices like FFR. Conventional coronary imaging may underappreciate the risk associated with long, mildly stenotic segments that, despite appearing less threatening, may harbor a similar pro-atherogenic and pro-thrombotic environment as focal high-grade lesions. This nuanced understanding encourages a more comprehensive evaluation that considers lesion geometry, flow patterns, and segmental hemodynamics. From a broader perspective, our results contribute to an evolving understanding that lesion vulnerability is not strictly dependent on anatomical severity. The comparable rheological conditions observed across lesion types imply that both focal and diffuse patterns can predispose to adverse outcomes, challenging the assumption that physiological or ischemic significance alone adequately captures lesion risk. Ultimately, this study shifts the focus from a binary interpretation of stenosis severity toward a more integrative, flow-centered view of coronary artery disease, with practical implications for clinicians aiming to optimize diagnostic accuracy and patient management.

### Limitations

Our study has limitations, including the relatively small number of LAD segments analyzed and the lack of comparison with an FFR group. However, the aim of our study was to compare the rheology of short versus long coronary lesions, not to compare rheology with FFR, which operates at a different level. Furthermore, the small sample size limited the statistical stability and generalizability of the observed associations between lesion morphology and local hemodynamics. Finally, we did not investigate differences in plaque composition or plaque burden, as these pertain to plaque vulnerability rather than stenosis rheology.

## 5. Conclusions

The results of our study indicate that long, angiographically mild coronary stenoses exhibit a coronary flow profile, in terms of WSS and RT, comparable to that of short, highly significant coronary stenoses. This similarity may explain why both types of lesions can possess similar physiological functional values.

## Figures and Tables

**Table 1:** Clinical and anatomical characteristics of the patient population. BMI: Body mass index; EDV: end-diastolic volume; ESV: end-systolic volume; LAD: left anterior descending. D.ref: Reference Diameter.

	Focal Significant Stenosis N = 16	Diffuse–Long Non-Significant Stenosis N = 14	*p*
Demographics			
Age (years)	53 [45–66]	55 [46–68]	0.82
Men, n (%)	10 (62.5)	9 (64.2)	0.92
BMI (Kg/m^2^)	27 [25–31]	27 [25–32]	0.65
Cardiovascular risk factors			
Hypertension, n (%)	12 (75.0)	11 (78.5)	0.82
Current Smokers, n (%)	4 (25.0)	3 (21.4)	0.81
Previous Smokers, n (%)	3 (18.7)	3 (21.4)	0.85
Baseline lipid profile			
Total cholesterol (mg/dL)	182.3 [166.2–209.5]	183.4 [167.6–211.8]	0.81
Triglycerides (mg/dL)	113.8 [86.9–162.3]	115.1 [89.3–165.1]	0.76
High-density lipoprotein (mg/dL)	40.3 [33.1–55.7]	42.3 [35.2–59.1]	0.79
Low-density lipoprotein (mg/dL)	119.4 [104.3–145.6]	118.0 [105.9–152.4]	0.86
Flow			
Hear Rate (bpm) at image acquisition	68.1 ± 7.5	69.2 ± 8.3	0.70
EDV (ml)	75.4 ± 18.2	74.3 ± 19.4	0.87
ESV (ml)	31.0 ± 7.6	30.2 ± 8.1	0.78
LAD anatomical characteristics			
LAD D.ref. (mm)	3.46 ± 0.7	3.47 ± 0.7	0.83
LAD stenosis diameter (%)	84.6 ± 4.3	42.9 ± 4.5	<0.001
LAD stenosis length (mm)	18.2 ± 1.1	38.9 ± 1.0	<0.001

## Data Availability

Data can be available upon request by contacting the corresponding author.

## Abbreviations

The following abbreviations are used in this manuscript:

CCTA	Computed Tomography Angiography
CFD	Computational Fluid Dynamic
FFR	Fractional Flow Reserve
LAD	Left Anterior Descending
RT	Resident Time
WSS	Wall Shear Stress
